# Thalidomide results in diminished ovarian reserve in reproductive age female IBD patients

**DOI:** 10.1097/MD.0000000000006540

**Published:** 2017-05-26

**Authors:** Xiang Peng, Min Zhi, Ming Wei, Ting-Ting Li, Min Zhang, Yuan-Qi Zhang, Huan He, Mingli Su, Wei Wang, Jun-rong Chen, Jian Tang, Xiang Gao, Pin-Jin Hu, Xiao-Yan Liang

**Affiliations:** a Department of Gastroenterology, the Sixth Affiliated Hospital, Sun Yat-sen University; b Guangzhou Daan Clinical Laboratory Centre Co. Ltd; c Department of Reproductive, the Sixth Affiliated Hospital, Sun Yat-sen University, Guangzhou, Guangdong Province, China.

**Keywords:** diminished ovarian reserve, inflammatory bowel disease, thalidomide

## Abstract

The effectiveness of thalidomide in treating inflammatory bowel disease (IBD) has been widely recognized. Meanwhile, many serious adverse drug reactions have been observed, but no know reports on ovarian reserve function.

Female patients, ranging in age between 18 and 40, were referred to our institution to undergo sex hormone detection and ultrasonic scanning for ovarian function assessment, between February 1, 2016 and September 31, 2016.

Thirty-three patients treated with thalidomide (group A), 73 patients without thalidomide (group B), and 78 healthy women as control were studied. Menstrual disorder was higher in group A than group B (78.8% vs 57.2%, *P* < 0.05), and both groups were higher than control group 33.3%, *P* < 0.05. Anti-Mullerian hormone (AMH) levels and antral follicle count (AFC) in group A were lower than group B, *P* < 0.05, while estradiol (E2) and follicle-stimulating hormone (FSH) levels were no different between 2 groups. Crohn Disease Endoscopic Index of Severity (CDEIS) and thalidomide were the independent risk factors in diminished ovarian reserve (DOR), and when dose reached 75 mg/day, 5 g total, or when treatment time reached 10 months respectively. These influence may increasing (*P* < 0.05), but they may recover after stopping (*P* < 0.05).

Thalidomide was an independent risk factor leading to DOR in female IBD patients, the influence may increasing when daily dose and accumulated dose reached 75 mg/day and 5 g total dose, but may be reversed by stopping.

## 1 Introduction

Thalidomide, its trade-name Contergan, is a dramatic medicine known for its effect on anxiety, insomnia, gastritis, and tension when used in West Germany in 1957. Afterwards, it was used against nausea and to alleviate morning sickness in pregnant women. Shortly after the drug was prescribed, about 10,000 cases were reported of infants with phocomelia due to thalidomide use worldwide. And due to its teratogenicity and neurotoxicity, it was withdraw from the clinic use.^[1]^ In 1965, Israeli dermatologist Sheskin found thalidomide to be beneficial in the treatment of erythema nodosum leprosy (ENL) occasionally.^[2]^ Later in 1998, thalidomide was approved in the United States by the Food and Drug Administration as therapy for ENL.^[3]^ Later research showed that it was used for number of conditions including erythema nodosum leprosum, multiple myeloma, various other cancers, and a number of skin conditions that have not responded to usual treatment.^[4]^ Recent research has found that thalidomide played an important role in treating inflammatory bowel disease (IBD).^[5–7]^ While, thalidomide may cause side effects, such as drowsiness, constipation, peripheral neuropathy, and so on.^[8–10]^ In addition to above-mentioned symptoms, we found that using thalidomide to treating IBD patient may resulted in menstrual disorders, such as menstruation reduction, irregular menstrual cycle, or longer menstrual cycle. These gender-dependent symptoms were speculated to be related to diminish ovarian reserve caused by using thalidomide. AMH is an important index to monitor the function of ovarian reserve, is majorly secreted by preantral follicles and antral follicles without fluctuation during menstrual cycle, not affected by hormone, and is affected earlier than sex hormones. Therefore, this study was to investigate IBD and the effect of thalidomide on function of ovarian reserve.

## 2 Methods

### 2.1 Ethical approval

The approval for conducting this study was granted by the Ethnic Committee of Sun Yat-sen University of Sixth Affiliated Hospital (2016ZSLYEC-067).

### 2.2 Patients and samples

Female IBD patients, aged 18 to 40 were recruited while visiting the Inflammatory Bowel Disease Center at the 6th Affiliated Hospital of Sun Yat-sen University from February 1, 2016 to September 31, 2016. The IBD diagnoses were confirmed by clinic symptoms, physical condition, endoscopy, radiology, and pathology. The study includes 33 IBD patients only taking thalidomide or thalidomide and other medicines more than 8 weeks. Seventy-three other patients, who never took thalidomide, used glucocorticoids, infliximab, azathioprine, methotrexate, full enteral nutrition, 5-aminosalicylic acid (5-ASA) or without drug treatment were also studied. The patients with the following were excluded: abnormal menstrual cycle or menstrual flow, ovarian function failure, the unrelated reproductive system diseases such as endocrine disorder, hysterectomy, pelvic radiotherapy, ovariectomy, polycystic ovary syndrome, endometriosis, ovarian granulosa cell cancer, taking birth control pills, and pregnancy 3 months before selection.

Healthy subjects were selected from healthy women; nonpregnancy, not taking birth control pills, and in similar age to the patient's group.

According to the evaluation of clinical diagnosis for patient's symptom, thalidomide or azathioprine was added as treatment for some of IBD patients and thalidomide was removed from the treatment of some other IBD patients.

### 2.3 Measure

#### 2.3.1 Collection of demography and clinic data

Age, extent, behavior, complication, Crohn Disease Active Index (CDAI) or Mayo, body mass index (BMI), hemoglobin, high-sensitivity C-reactive protein (Hs-CRP), erythrocyte sedimentation rate (ESR), platelet, albumin; menstruation situation, including cycle and flow. Normal menstruation includes the cycle of 25 to 35 days, menstrual period of 2 to 7 days, and menstrual flow of 20 to 60 mL. Having any or more abnormal menstrual indexes is abnormal is defined as a menstrual disorder. Amenorrhea is defined as when regular menstruation stops longer than 6 months or original own menstrual cycle stops more than 3 cycles. Thalidomide usages, such as daily and accumulative dosages, and combination with other therapeutic methods, were recorded.

#### 2.3.2 Method to evaluate the DOR

To measure AMH levels, whole blood samples were taken from the vein of IBD patients and healthy subjects. AMH level was determined by enzyme-linked immunosorbent assay (ELISA). AMH ≤ 1.1 μg/L was defined as DOR.^[11,12]^ AFC, was evaluated by using transvaginal, 3-dimensional color Doppler ultrasonography (Voluson 730 Expert, GE, Kretztechnik, Austria, 8 MHZ frequency) on third day of menstrual cycle. The foundational follicles at 5 to 10 mm on each ovary were determined and the sum of antral follicles from ovaries is determined to be the AFC. All of the ultrasonography was performed by a single clinician. FSH and E2 levels were determined by microparticle enzyme immunoassay on the third day of menstrual cycle.

### 2.4 Statistical analysis

SPSS16.0 software was used in statistics. X ± S is used to show the normal distribution of data; variance analysis or *T* test is adopted in normal distribution between many group data and α = 0.05 is the inspection standard. Median is used to show nonnormal distribution of data. Percentage showed quantitative data; chi-square test and Fisher precise probability method were used to show difference significance between group data. Using a scatter plot analysis mode, logistics analysis of univariate and multivariate of correction for confounding factors was used for the correlation between AMH level and other factors such as dose, accumulated dose, usage time of thalidomide, *P* < 0.05 as statistical significance.

## 3 Results

### 3.1 Comparison of clinical information of IBD patients between using thalidomide and without using thalidomide

IBD patients in this study were divided into 2 groups as using thalidomide (33 cases) and not using thalidomide (73 cases). The related clinical information in 2 groups of patients were compiled and compared. The related clinical information included Crohn disease (CD), ulcerative colitis (UC), extent, behavior, complication, active index, Hs-CRP, ESR, platelet count, BMI, hemoglobin value, albumin levels, and CDEIS. These clinical criteria were evaluated by comparing them between 2 groups. *P* values were calculated by using *T* test. None of the above criteria were found significant between the 2 groups (*P* > 0.05). The results showed that the thalidomide treated and nontreated groups did not have significant difference in any observed clinical criteria (Table 1).

**Table 1:**
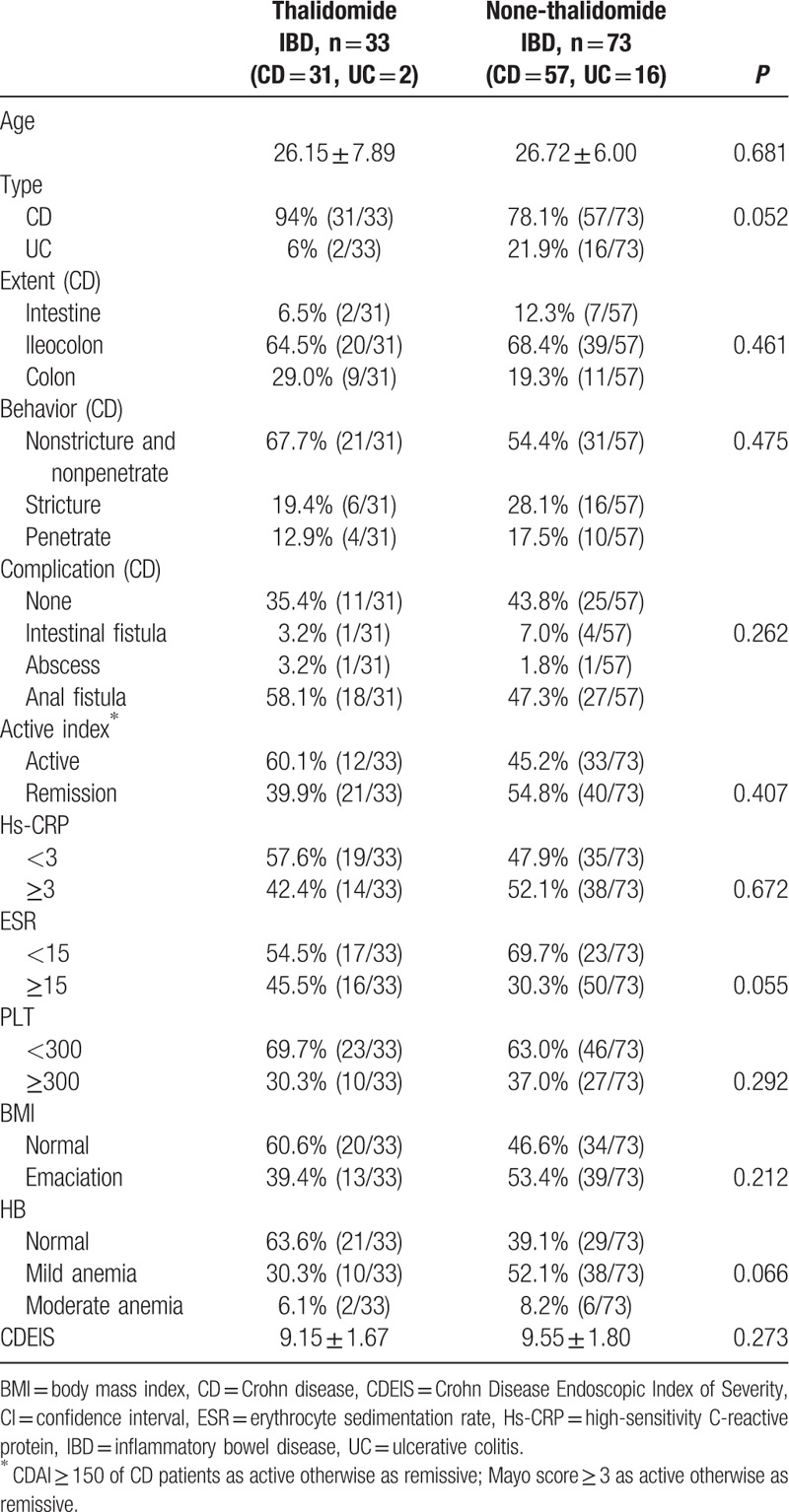
Demography and clinic information of female IBD patients taking and nontaking thalidomide.

### 3.2 Menstruation situation

The menstruation cycle has three situations: normal, disorder, and amenorrhea. The menstruation situations of study subjects were recorded according to the description given by subjects when the study a started. One IBD patient from the group using thalidomide had amenorrhea accounting for 3% (1/33) whereas no IBD patient from not using thalidomide had amenorrhea as 0% (0/73). The menstrual disorder was also counted as described by IBD patients. Twenty-six out of 33 patients claimed to have menstrual disorder, which accounted for 78.8%. The menstrual disorder (78.8%) in the patients using thalidomide was higher than in the patients not using thalidomide (57.5%, 42/73). The difference was significant *P* = 0.035. In healthy group, no amenorrhea was found (0/78) and menstrual disorder was 33.3% (26/78), which was lower than in the IBD patient (*P* = 2.5 × 10^–5^).

### 3.3 The AMH levels in the patients using and not using thalidomide, and healthy subjects

AMH level is used as an indicator for ovarian reserve function. AMH levels in all of study projects were measured. If an AMH level was lower than 1.1 μg/L, the ovarian reserve is defined as DOR. The ovarian reserve was considered as normal ovarian function when AMH level was greater than 1.1 μg/L. The AMH in IBD patients not using thalidomide (2.68 ± 2.06 μg/L) was lower than that of healthy subjects (5.61 ± 5.06 μg/L), *P* = 4.80 × 10^–6^, but higher than that of IBD patient using thalidomide (0.85 ± 0.81 μg/L), *P* = 2.42 × 10^–9^. Age factor affecting AMH was also evaluated in the study. Since AMH level decreased as age increased,^[13]^ the subjects in the study were divide into 2 groups: above and below the age of 30. Correlation analysis of age and ovarian reserve used 30 years old as group cutoff (<30 and ≥30) to compare the group of using thalidomide with the group not using thalidomide, and with the healthy group (*P* = 6.25 × 10^–10^, *P* = 0.001) (Table 2). From the data of AMH levels, it was found that using thalidomide is the major factor affecting the AMH level or ovarian reserve function in IBD patients although age factor also resulted in the decrease of AMH level in IBD patients.

**Table 2:**
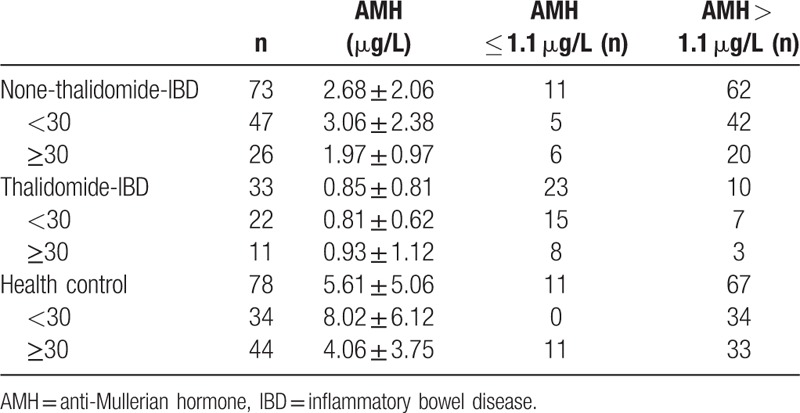
Comparison of AMH of thalidomide intake with that nonthalidomide intake and with that of healthy subjects in different age groups.

### 3.4 Analysis of single risk factor of AMH level in IBD patients and control subjects

In order to exclude any clinical factor that might not result in lower AMH level, single factor logistics analysis was used to determine whether any clinical factor, such as age, IBD type, extent colon type, behavior, complication, thalidomide usage, active index, HS-CRP level, ESR level, platelet level, hemoglobin, albumin, CDEIS, and BMI, significantly caused lower level of AMH level. If *P*-value is less than 0.1, the factor was considered as clinical symptom that caused decrease of AMH in IBD patients. In all of clinical factors, IBD types, intestinal fistula, and anal fistula complications had α values 0.094, 0.092, and 0.055, respectively, which were less than 0.1. The results also showed that CDEIS was the clinical factor significantly causing an increased number of patients with AMH level ≤ 1.1 μg/L (*P* = 0.036). The usage of thalidomide as a clinical factor was evaluated and yielded much smaller *P*-value 2.23 × 10^–4^. These results showed that the usage of thalidomide is a major factor greatly reducing the level of AMH in IBD patients (Table 3).

**Table 3:**
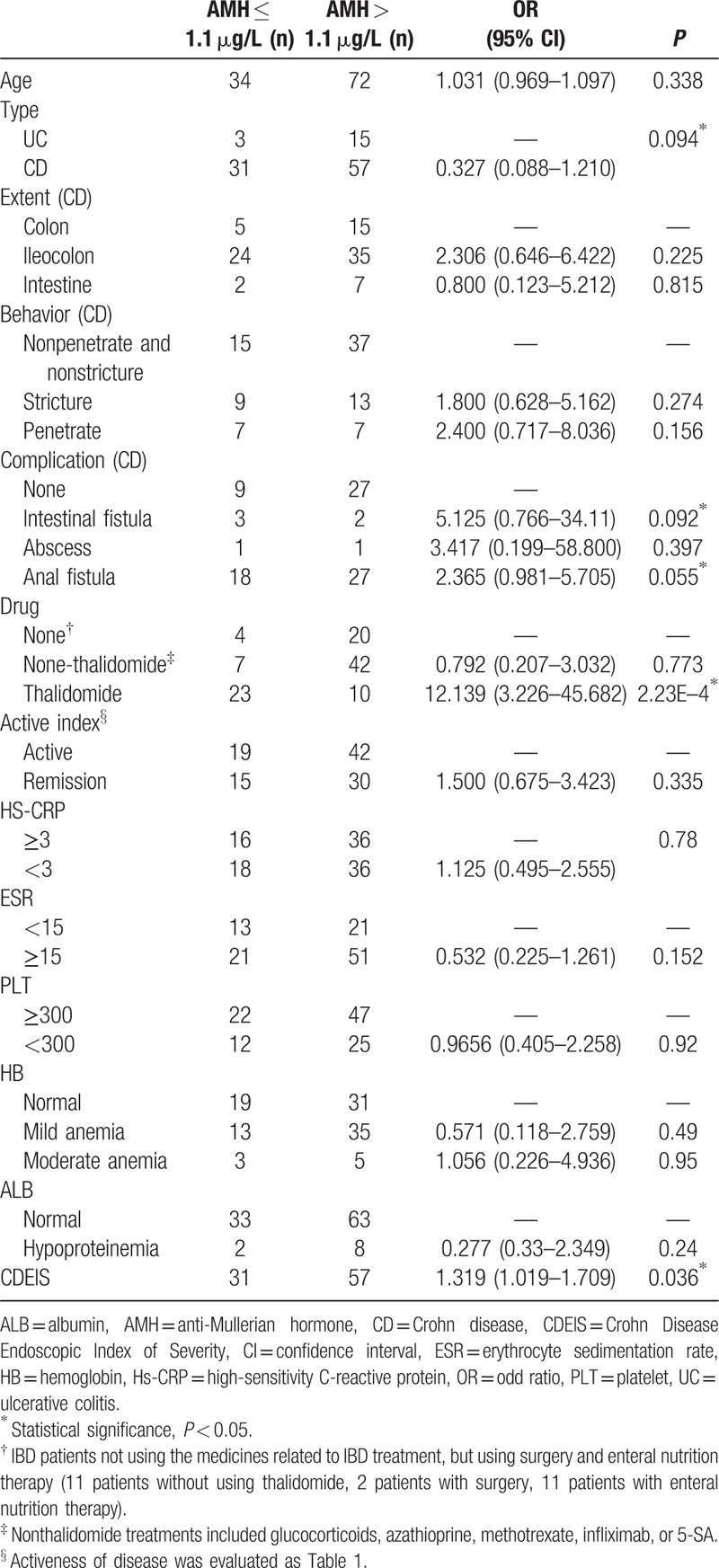
Univariate analysis of IBD patients with low level of AMH.

### 3.5 Multivariate analysis of risk factors previously identified by the univariate analysis

The risk factors affecting AMH level, which were identified by the univariate analysis, were subjected to the multivariate analysis. The evaluation of multivariate analysis was determined by *P*-value < 0.05. In univariate analysis, IBD type, intestinal fistula, and anal fistula complications were identified as the risk factors with *P* < 0.1. Multivariate analysis results showed that these factors were not the clinical factors affecting AMH level. The *P* values were respectively as 0.932, 0.303, and 0.219. CDEIS and using thalidomide when analyzed together by multivariate analysis were identified as significant effectors causing the increased number of patients with lower AMH level ≤ 1.1 μg/L (*P* = 0.036 and 2.23 × 10^–4^). This result demonstrated that CDEIS and the usage of thalidomide as reaction-stopper were independent risk factors leading to the increase of IBD patients with lower level of AMH (Table 4).

**Table 4:**
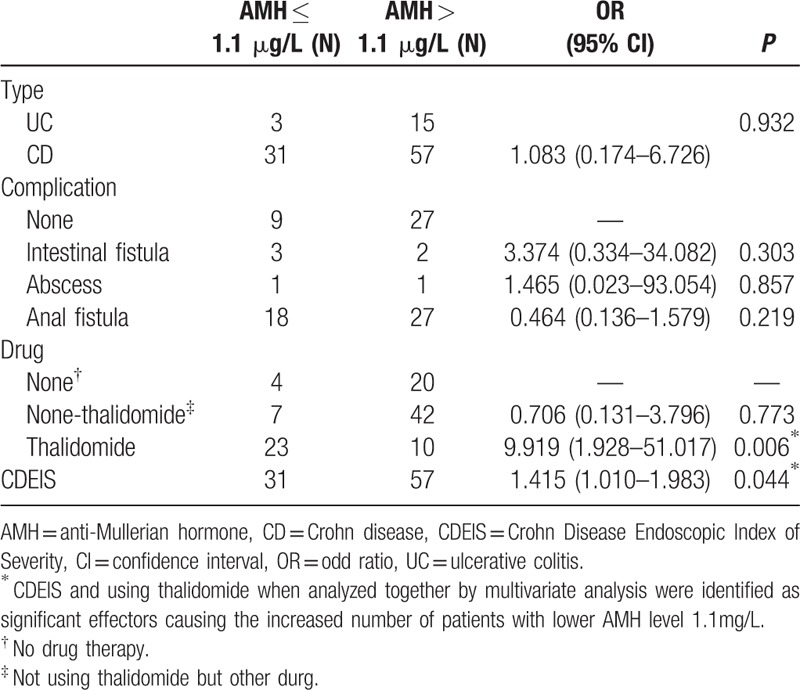
Multivariate analysis of the decreased level of AMH in female IBD patients.

### 3.6 AMH changes before and after using thalidomide or azathioprine as the treatments in IBD patients

**Figure 1:**
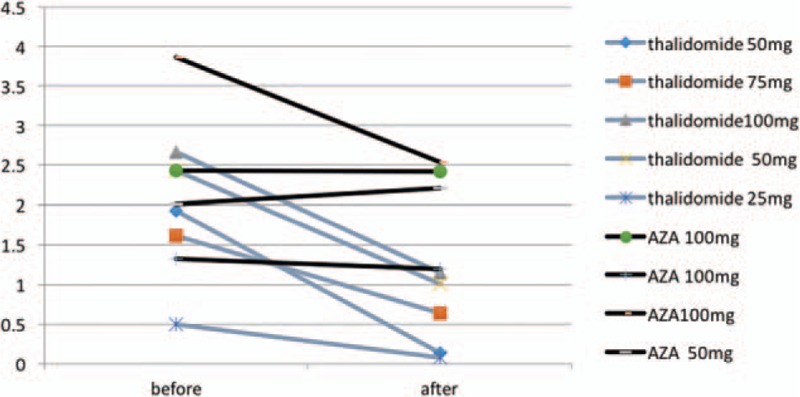
AMH changes before and after using thalidomide or AZA = azathioprine.

AMH levels in 5 patients using thalidomide (2 at 50 mg, 1 at 100 mg, 1 at 75 mg, 1 at 25 mg) and 4 patients using azathioprine (3 at 100 mg, 1 at 50 mg) were determined before using medications and for 3 months after using medications. The average AMH level in 4 patients before using azathioprine was 2.41 ± 1.08 μg/L. After 3 months using azathioprine, the average AMH level slightly decreased to 2.10 ± 0.61 μg/L. *T* test showed no significance changes with *P* = 0.428. The average AMH level in the 5 patients before starting thalidomide treatment was at the normal level of 1.85 ± 0.85 μg/L. After using thalidomide for 3 months the average AMH level dramatically decreased to 0.604 ± 0.49 μg/L with *P* = 0.007 (Fig. 1). The result demonstrated that using thalidomide is the major factor causing DOR rather than other medication such as azathioprine.

### 3.7 Recovery of AMH level in patients after stopping thalidomide usage

The dosage used in 7 patients (ages 19–30 years old) reached either a daily dose > 75 mg or accumulative dose > 5 g, which significantly reduced the levels of AMH. Furthermore, the average level of AMH in 7 patients before stopping thalidomide treatment was 0.628 ± 0.72 μg/L. The AMH levels of 5 patients were lower than 1.1 μg/L. The 7 patients then stopped thalidomide treatment. After 3 months of stopping thalidomide treatment, the AMH level in 3 out of 5 patients with AMH < 1.1 μg/L recovered to greater than 1.1 μg/L. The average AMH level in 7 patients was 14.409 ± 23.39 μg/L. The overall increase of average AMH level in 7 patients stopping thalidomide treatment for 3 months was significant, comparing with before stopping thalidomide treatment, with *P* = 0.044 (Table 5).

**Table 5:**
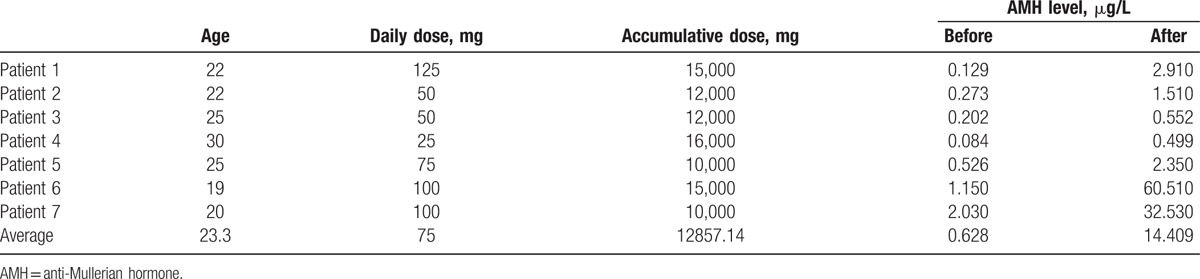
Recovery of AMH level after stopping thalidomide treatment.

### 3.8 The relationship between thalidomide dose, accumulative dose, prescription time with AMH

**Figure 2:**
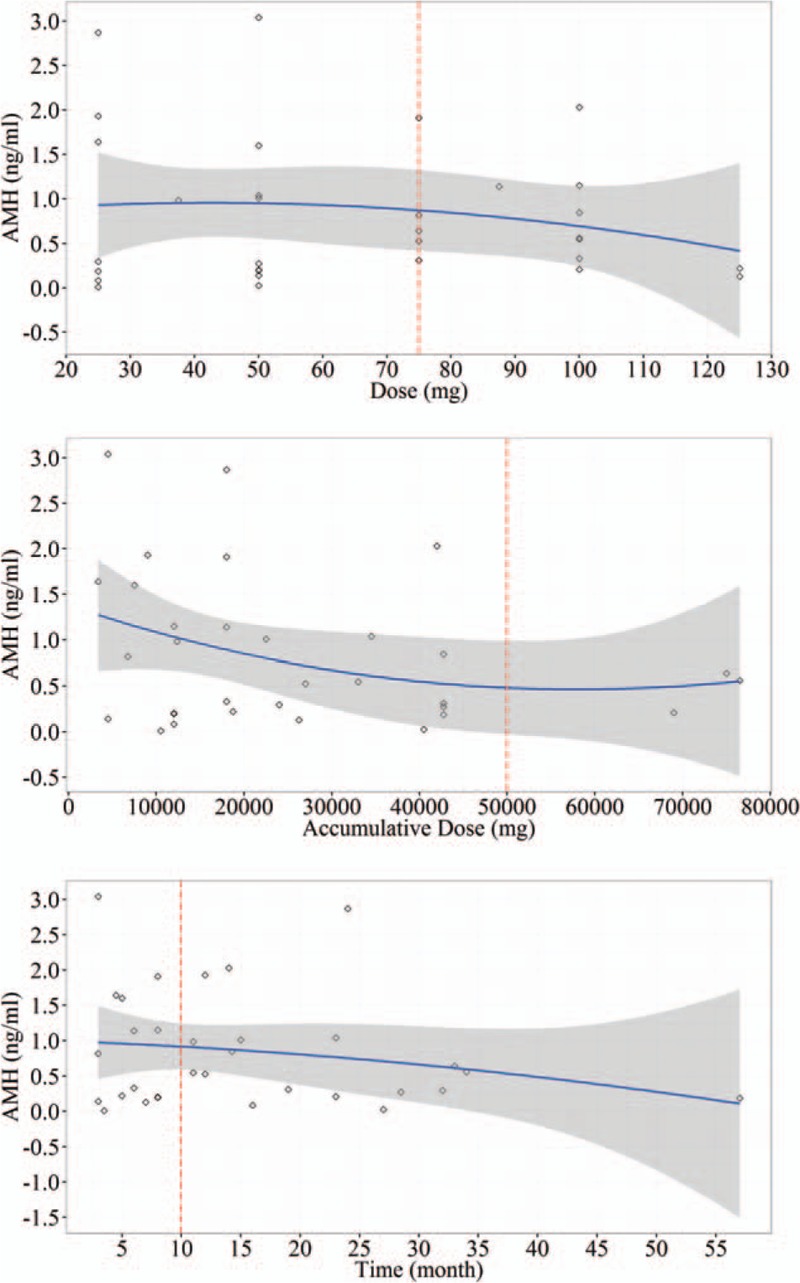
The relationship between thalidomide dose, accumulative dose, prescription time with AMH.

When IBD patients were treated with thalidomide, 2 factors, dose and prescription time might play key roles causing decreasing AMH level. The effects of daily dose, accumulative dose, and prescription time on ovarian reserve function on patients were determined by AMH level of patients. In all of thalidomide treated patients, AMH level showed a notable decrease when either daily treatment dose or accumulative treatment dose reached 75 mg or 5 g, respectively (*P* < 0.05) (Fig. 2A and B). In addition to dosage effects, the prescription time (thalidomide treatment time) >8 months also caused the decrease of AMH level (Fig. 2C). These results concluded that daily dose, accumulative dose, and prescription time could result in DOR in IBD patients.

### 3.9 Effect of thalidomide treatment on antral follicle count (AFC) in IBD patients

AFC is a method of transvaginal ultrasound used to measure ovarian reserve function or remaining egg supply. Since thalidomide treatment led to DOR (lower level of AMH), AFC, as a complementary method, was used to observe the function of ovaries through clinical diagnosis. AFCs were performed on 21 IBD patients, including 14 patients without thalidomide treatment and 7 patients with thalidomide treatment, by means of transvaginal ultrasound. It was found that AFC in 7 patients treated with thalidomide was obviously lower (6 ± 3) than in 14 patients without thalidomide treatment (12 ± 7) (Table 6). *T* test analysis showed that the decrease of AFC in the patients with thalidomide treatment was significant when comparing with AFC in the patients without thalidomide treatment (*P* = 0.016) (Table 6). From these results, it demonstrated that not only AMH level was decreased through thalidomide treatment but also did potential of ovary to produce egg.

**Table 6:**
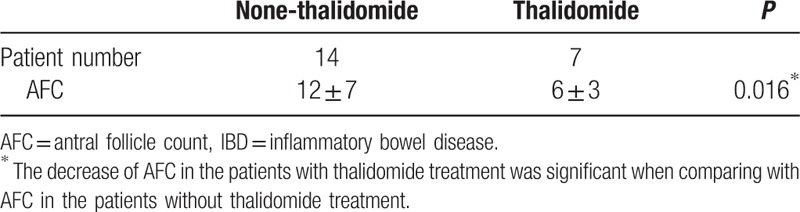
Effect of thalidomide treatment on AFC in IBD patients.

### 3.10 Effects of thalidomide treatment on female hormones in IBD patients

E2 and FSH are female hormones that are related to female menstrual cycle and ovarian reserve function. Therefore, E2 and FSH levels were important criteria to be observed during treatment of IBD for female patients. It has shown that AMH and AFC were affected significantly by thalidomide treatment in this study. Furthermore, E2 and FSH levels were observed in 18 patients without thalidomide treatment and 26 patients with thalidomide treatment. The average E2 level in 26 patients treated with thalidomide was slightly lower (69.78 ± 64.37) than in patients without thalidomide treatment (82.61 ± 74.25). The change was not significant via *T* test (*P* = 0.557). The average FSH level in 26 patients treated with thalidomide was 10.64 ± 14.57 while the average FSH level in 18 patients without thalidomide treatment was measured as 4.42 ± 2.27. Although FSH levels in thalidomide treatment group were higher, the *T* test showed that *P*-value was greater than 0.05 (Table 7). These results showed that E2 and FSH might not be affected by thalidomide treatment in IBD patients.

**Table 7:**
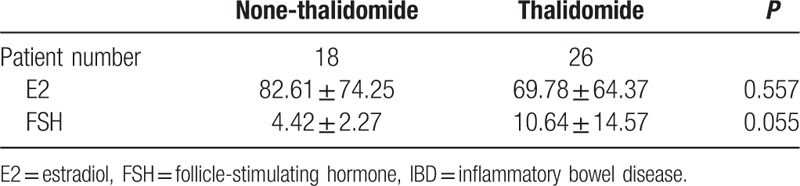
Effects of thalidomide treatment on sex hormones in IBD patients.

## 4 Discussion

Thalidomide is a synthetic derivative of glutamic acid, which was first synthesized in Western Germany as a sedative widely used for treating insomnia and effects of pregnancy.^[14]^ In 1960 it was pulled out of the market due to that it caused neonatal malformations.^[1]^ In 1965, Israeli dermatologist Sheskin used thalidomide to treat ENL and obtained satisfactory results.^[2]^ Recent studies showed that the role of thalidomide in immunomodulatory, antiinflammation, and antiangiogenesis was emphasized again.^[4,15]^ These factors led to thalidomide's use in treating malignant cancer and rheumatic connective tissue diseases. Recent study also found that thalidomide played an important role in treating refractory inflammatory bowel disease (IBD).^[5–7]^ Thalidomide as an immunosuppressive agent, inhibited mononuclear cell factor to play the role of immunosuppression and stimulated T lymphocyte activation to activate immunoreaction, its antiinflammatory mechanism is to inhibit the release of tumor necrosis factor-α (TNF-α) by inhibiting synthesis of TNF-α, which led from promoting degradation of TNF-α mRNA.^[16–18]^ Waters et al^[19]^ first reported that thalidomide might effectively treat refractory UC in 1979. In 1997, Wettstein et al^[20]^ reported that 1 case of refractory CD obtained symptom relief by treatment with thalidomide, and later the studies of thalidomide used in IBD treatment were repeatedly reported. Two open clinical trials of treating refractory CD with thalidomide showed that clinical response rates were respectively 64% and 70% in 12-week treatment.^[8,21]^ Reviewing 12 cases of refractory CD with different treatments, Plamondon et al^[5]^ found that azathioprine, methotrexate, and anti-TNF-α agents were ineffective but 100 mg/day of thalidomide treatment for 12 months resulted in 75% of average response rate and CD activity index (CDAI) decrease 212 points. Comparing with other immunoinhibitor, thalidomide took effect at 2 to 4 weeks after stating treatment^[22]^ and showed more benefit for controlling the acute symptom due to its rapid response time. Teratogenicity is the most widely known side effect of thalidomide. Another common side effect was peripheral nerve lesions with symptoms including peripheral paresthesia, limb numbness, drowsiness, constipation, headache, dizziness, and so on.^[8–10]^ While analyzing 10,456 patient cases who used thalidomide in USA, it was found that within the first 18 months, occurrence rates of drowsiness, rash, weakness, and peripheral paresthesia were respectively 1.8%, 1.4%, 1.2%, and 0.67%.^[21]^ Felipez et al^[7]^ reported 42% the cases exhibited peripheral nerve lesions, 8% with dizziness, 8% with allergy. Other reports found that 50% of patients with using thalidomide had peripheral nerve lesions but symptom in most of patients were moderate and reversible.^[22]^ Fewer adverse reaction cases were reported when using thalidomide to treat lupus erythematosus, rheumatoid arthritis, and multiple myeloma to cause amenorrhea of patients.^[23,24]^ In various studies and reports, 32 cases leading to amenorrhea of patients in an average of 6 months when beginning thalidomide treatment occur (which have range of 1–42 months).^[24]^ The clinic indexes used to evaluate ovary reserve function included sex hormone (E2, FSH), AFC, and AMH.^[25]^ Sex hormone and AFC measurements had certain limitations such as subjective factors relating to diagnostic instrument operators and pelvic environment (including infection and active bowl movements) affecting ultrasound image, and small primary oocytes resulting in unclear ultrasound image. These factors may led to errors in antral follicle cell counts. E2 and FSH existing in the same the neuroendocrine system axis. They are regulated by multihormones feedback loops and affected by follicle cycle. Differing from these index factors, AMH as a member of transforming growth factor-β (TGF-β) was secreted by preantral and antral follicles and was not affected by menstrual cycle and hormones, AMH's change was also earlier than sex hormone, and tests for AMH were convenient. Therefore, this study used AMH as major evaluation factor for ovarian reserve function.

By following the menstruation cycles of IBD patient, the incidence of menstrual disorders in thalidomide treatment patients was much higher than in nonthalidomide treatment patients, but there was no report about the effect of thalidomide on ovarian reserve function. Our study found that ovarian reserve in IBD patients without thalidomide treatment was lower than in healthy subjects but much higher than in IBD patients with thalidomide treatment. Continuous use of thalidomide may lead to further decline of ovarian reserve function.

Our result demonstrated the significant difference of AMH values between IBD patients and healthy subjects of the same age (see Table 2), and IBD patients showed the lowest level of AMH. Although sex hormone levels were not related to thalidomide treatment, AFC in IBD patients with thalidomide treatment was lower than IBD patients without thalidomide treatment. It was considered that thalidomide treatment might lead to further decrease of ovarian reserve function in female IBD patients. AMH was much more sensitive in predicting ovarian reserve function because its change was earlier than FSH and E2. Logistical analysis was used to identify certain risk factor leading to the decrease of ovarian reserve function in IBD patients. We discovered that thalidomide was major risk factor, causing decreased AMH. The results also showed the diminish ovarian reserve function of patients was not related to CD type, extent, behavior, complication, inflammation activity, nutritional status, and also not related with other nonthalidomide drugs, such as immunosuppressant or infliximab, which were consistent with Khamashta's investigation stating that azathioprine, cyclosporine, and methotrexate were not related to ovarian failure.^[26]^ In order to further identify the effect of thalidomide on ovarian reserve function, we compared AMH measurements pre- and post- (3 months) thalidomide or azathioprine treatment. We found that AMH levels of IBD patients with 3 months of thalidomide treatment decreased notably whereas AMH level of patients with treatment of azathioprine remained unchanged (Fig. 1). Meanwhile, as the ovarian reserve function gradually recovered after stoppage of thalidomide after 3 months (Table 5) and it was implied that effect of thalidomide on ovarian reserve function was reversible. Due to smaller sample number in present study, we are recruiting more patients in the future study to prove the reversibility of effect of thalidomide on ovarian reserve function.

Correlation analysis of thalidomide treatment and DOR found that a single dose greater than 75 mg, accumulated dose beyond 5 g, or treatment time over 10 months resulted in decrease of AMH levels in IBD patients. These data mean that the toxicity of thalidomide to ovary was related to the daily dose, accumulated dose, and treatment time (Fig. 2).

Our study revealed that thalidomide treatment might led to the further damage of ovarian reserve function in IBD patients with lower ovarian reserve function. The mechanism leading to disease in female IBD patients resulting in DOR still unknown. We presume that thalidomide might act on transforming growth factor (TGF) pathway to affect follicular cell development. Transforming growth factor-α (TGF-α) may promote cell growth and transformation of normal cells. In vitro studies confirmed that TGF-α, which was localized and expressed at ovarian follicles and stroma interval, has a regulatory role on ovarian tissue during entire procedure of growth, degeneration, and atresia of follicular.^[27,28]^ TGF-α was highly expressed in oocytes during primordial follicles stage, then the expression of TGF-α gradually decreases along with maturation of follicles, which implies that TGF-α synthesized in early stage of follicle formation might participates in development of primordial follicles and early growth of oocytes.^[29,30]^ Thalidomide not only plays an immune-inhibitory role for TNF-α, interleukin 6 (IL-6), and interleukin 8 (IL-8) but also an inhibitory role for TGF-α and TGF-β.^[31]^ Therefore, thalidomide-treated patients might encounter inhibited expressions of TGF-α and TGF-β, further affecting the development of follicles, leading to diminish ovarian reserve. These hypotheses need further investigation and research.

Ovarian reserve function represents women gametogenesis and the ability to produce steroid hormones, which reflects fertility potential and is related to menopause age. Ovarian reserve decrease is stated as the reduced number of raised follicles and/or the poor quality of oocytes, which caused decrease of fertility and early menopause. IBD incidence in worldwide has increased significantly and became a global disease.^[32,33]^ Since most of the IBD patients are young females,^[34–36]^ clinical problems such as fertility, pregnancy, drug safety, and breast-feeding became prominent and attracted the great attention. Thalidomide is low cost treatment and many clinic studies show that it provides the benefit for refractory IBD and for patients not responding immunosuppressant. Thalidomide can be a good alternative drug, however its side effects, especially its adverse reactions easily ignored in clinics, should be closely monitored during its use. As most of IBD patients are young female, the DOR will seriously affect their fertility and life style if the function is lost to cause amenorrhea. Decrease of ovarian reserve function and the toxic side effects rise with increased thalidomide dosage, accumulate dosage, prescription time. Therefore, the function of ovarian reserve in patients should be closely monitored when thalidomide is used to treat IBD. When thalidomide daily and accumulated dosage are greater than 75 mg and 5 g and treatment period is longer than 10 months, thalidomide treatment should be used along with careful evaluation of patient situation or replaced by other immunosuppressant and biologics with less effect on ovarian reserve. This will help to prevent early exhaustion of ovary function, which may affect quality of IBD patient life.

## 5 Conclusion

Since thalidomide was not primary choice of medications for IBD patients, most of IBD patients received infliximab, glucococrticoid, or azathioprine treatment. Therefore, the number of patients treated with thalidomide was relatively small, especially after dividing the patients into before and after treatment groups. Another limiting situation was that most of patients receiving thalidomide treatment for relatively short time, made it not possible to monitor long-term effects of thalidomide on ovarian reserve function of IBD patients. The available number of IBD patients stopping thalidomide treatment on pregnancy or on infant deformity is not observed. Although thalidomide is not primary choice for treating IBD, more reports have confirmed the efficacy of thalidomide in treating refractory IBD. Our study revealed that thalidomide treatment for IBD resulted in DOR for reproductive period women. The ovarian reserve function of female IBD patient should be closely monitored when thalidomide is used to treat IBD.

## Acknowledgment

We deeply appreciate the staff in 6th Hospital of Sun Yat-sen University for collecting cases.
